# Supplementary material for: The values and meanings of social activities for older urban men after retirement
Source: PLoS One. 2020 Nov 25;15(11):e0242859. doi: 10.1371/journal.pone.0242859 (PMC7688116; doi:10.1371/journal.pone.0242859)
Supplement: Attachment — Submitted filename: Response_to_Reviewers.docx [file pone.0242859.s001.docx]

Dear Editor and Reviewers,

Thank you very much for providing us with your insights regarding our manuscript. We have revised our paper accordingly, as shown point-by-point below. The additions are marked in red and deleted text is tracked via the “Track Changes” function in Microsoft Word. The line number in this document is based on the main manuscript with the revision history and revisions shown in-line.

We would appreciate it if you reviewed our manuscript again. Thank you very much.

**Responses and revisions regarding the suggestions from editor**

| **No.** | **Suggestions** | **Responses and revisions** |
| --- | --- | --- |
| 1 | Please ensure that your manuscript meets PLOS ONE's style requirements, including those for file naming. The PLOS ONE style templates can be found at  <https://journals.plos.org/plosone/s/file?id=wjVg/PLOSOne_formatting_sample_main_body.pdf> and  <https://journals.plos.org/plosone/s/file?id=ba62/PLOSOne_formatting_sample_title_authors_affiliations.pdf> | We have checked that our file meets PLOS ONE's style requirements and the file name of Fig. 1 was revised as follows.  Before revising: “20200708 Figure 1 350”  After revising: “Fig1.tif” |
| 2 | We note that you have indicated that data from this study are available upon request. PLOS only allows data to be available upon request if there are legal or ethical restrictions on sharing data publicly. For information on unacceptable data access restrictions, please see <http://journals.plos.org/plosone/s/data-availability#loc-unacceptable-data-access-restrictions>.  In your revised cover letter, please address the following prompts:  a) If there are ethical or legal restrictions on sharing a de-identified data set, please explain them in detail (e.g., data contain potentially identifying or sensitive patient information) and who has imposed them (e.g., an ethics committee). Please also provide contact information for a data access committee, ethics committee, or other institutional body to which data requests may be sent.  b) If there are no restrictions, please upload the minimal anonymized data set necessary to replicate your study findings as either Supporting Information files or to a stable, public repository and provide us with the relevant URLs, DOIs, or accession numbers. Please see <http://www.bmj.com/content/340/bmj.c181.long> for guidelines on how to de-identify and prepare clinical data for publication. For a list of acceptable repositories, please see <http://journals.plos.org/plosone/s/data-availability#loc-recommended-repositories>.  We will update your Data Availability statement on your behalf to reflect the information you provide. | Thank you for your detailed explanation of data availability. Our interview data contains potentially identifying and sensitive participants’ information. Therefore, we think there is an ethical limit to sharing the data publicly. Therefore, we have made the data available upon request. In the cover letter, we added the revised statement. Could you please update the “Data availability” section as follows? Thank you.  Data availability  Before revising: “The data for this study consists of transcripts of interviews with 15 participants and contain identifying items, and are therefore sensitive to privacy issues. As participants only allowed the interviews under promise of anonymity, we are expressly forbidden by the participants to make the full content of the interviews public. Anonymized excerpts from the full transcripts can be made available to qualified researchers by request to the ethical committee of the Faculty of Health Sciences, Hokkaido University, who can be contacted at shomu@hs.hokudai.ac.jp.”  After revising: “Our interview data contains potentially identifying and sensitive participants’ information. Therefore, there is an ethical limit to sharing the data publicly. Anonymized excerpts from the full transcripts can be made available to qualified researchers by request to the ethical committee of the Faculty of Health Sciences, Hokkaido University, who can be contacted at shomu@hs.hokudai.ac.jp.” |
| 3 | Thank you for stating the following in the Financial Disclosure section:  “This work was supported by JSPS KAKENHI [grant number JP18H03103]. In addition, we received support from Nihon Unisys, Ltd. for research expenses. The former sponsorad had no role in study design, data collection and analysis, decision to publish, or preparation of the manuscript. A researcher from Nihon Unisys, Ltd. helped collect interview data.”  We note that you received funding from a commercial source: “Nihon Unisys, Ltd”  Please provide an amended Competing Interests Statement that explicitly states this commercial funder, along with any other relevant declarations relating to employment, consultancy, patents, products in development, marketed products, etc.  Within this Competing Interests Statement, please confirm that this does not alter your adherence to all PLOS ONE policies on sharing data and materials by including the following statement: "This does not alter our adherence to PLOS ONE policies on sharing data and materials.” (as detailed online in our guide for authors http://journals.plos.org/plosone/s/competing-interests).  If there are restrictions on sharing of data and/or materials, please state these. Please note that we cannot proceed with consideration of your article until this information has been declared.  Please include your amended Competing Interests Statement within your cover letter. We will change the online submission form on your behalf.  Please know it is PLOS ONE policy for corresponding authors to declare, on behalf of all authors, all potential competing interests for the purposes of transparency. PLOS defines a competing interest as anything that interferes with, or could reasonably be perceived as interfering with, the full and objective presentation, peer review, editorial decision-making, or publication of research or non-research articles submitted to one of the journals. Competing interests can be financial or non-financial, professional, or personal. Competing interests can arise in relationship to an organization or another person. Please follow this link to our website for more details on competing interests: http://journals.plos.org/plosone/s/competing-interests. | Regarding financial competing interests, we received an unrestricted research grant from Nippon Unisys, Ltd. As for the funder’s role in the study design, a researcher from Nihon Unisys jointly collected data.  We do not have any other competing interests related to employment, consultancy, patents, products in development, marketed products, etc.  We added this information to the cover letter.  Could you update the “Competing Interests” section as follows? Thank you.  Competing Interests  Before revising: “The authors have declared that no competing interests exist.”  After revising: “This study received unrestricted research grants from Nihon Unisys, Ltd. A researcher of Nihon Unisys, Ltd. played a role in data collection. This relationship does not alter our adherence to PLOS ONE policies on sharing data and materials.” |
| 4 | PLOS requires an ORCID iD for the corresponding author in Editorial Manager on papers submitted after December 6th, 2016. Please ensure that you have an ORCID iD and that it is validated in Editorial Manager. To do this, go to ‘Update my Information’ (in the upper left-hand corner of the main menu), and click on the Fetch/Validate link next to the ORCID field. This will take you to the ORCID site and allow you to create a new iD or authenticate a pre-existing iD in Editorial Manager. Please see the following video for instructions on linking an ORCID iD to your Editorial Manager account: https://www.y outube.com/watch?v=_xcclfuvtxQ | We have linked the ORCID iDs to the Editorial Manager account for all authors who have an ORCID iD. |

**Responses and revisions regarding the suggestions from reviewer: 1**

| **No.** | **Suggestions** | **Responses and revisions** |
| --- | --- | --- |
| 1 | Page 1, the authors’ name. The last letter of the authors’ family name may be unnecessary. For example, Takashimaa should be replaced to Takashima. | Thank you for finding it. It was revised as follows:  Page 1, line 6  Before revising: “Takashimaa”  After revising: “Takashima” |
| 2 | Page 4, paragraph 3. I do not understand why you needed to show the information regarding Men’s Sheds here. To me, this specific information does not fit in the Introduction. You could add this kind of information as an example in the Discussion where you mentioned this topic. | Following your suggestion, the paragraph on Men’s Sheds was removed from the Introduction as follows:  Page 4, line 69 – page 5 line 80  Before revising: “…. participation in social activities in the community may help them adjust to additional free time.  One of the pioneering social activities in the community for men is Men’s Sheds, which is becoming popular internationally [13]. Men’s Sheds are usually community spaces where older men can interact while participating in various woodworking and other activities [14]. Men’s Sheds provide an unstructured environment for male fellowship and valuable activities [15]. In other words, Men’s Sheds have a positive impact on the health and well-being of older men by providing a space where valuable activities take place [16]. Previous studies on Men’s Sheds have reported the effects of providing a comfortable place in the community for older men rather than offering social activities themselves. However, the values and meanings of social activities themselves for older men have rarely been investigated.  Recently, the Japanese government has been promoting social participation….”  After revising: “…. participation in social activities in the community may help them adjust to additional free time.  ~~One of the pioneering social activities in the community for men is Men’s Sheds, which is becoming popular internationally [13]. Men’s Sheds are usually community spaces where older men can interact while participating in various woodworking and other activities [14]. Men’s Sheds provide an unstructured environment for male fellowship and valuable activities [15]. In other words, Men’s Sheds have a positive impact on the health and well-being of older men by providing a space where valuable activities take place [16]. Previous studies on Men’s Sheds have reported the effects of providing a comfortable place in the community for older men rather than offering social activities themselves. However, the values and meanings of social activities themselves for older men have rarely been investigated.~~  Recently, the Japanese government has been promoting social participation….”  In contrast, some descriptions about Men's Sheds in the Introduction were added to the Discussion as follows:  Page 35, line 600 – page 36, line 609  Before revising: “Men’s Sheds have demonstrated a certain effect as a place for social activities for older men in the community [24]. As men tend to be less involved in community activities than women, Men’s Sheds are a place where older men are more likely to participate in social activity in communities.”  After revising: “One of the pioneering social activities in the community for men is Men’s Sheds, which is becoming popular internationally [61]. Men’s Sheds have demonstrated a certain effect as a place for social activities for older men in the community [20]. Men’s Sheds are usually community spaces where older men can interact while participating in various woodworking and other activities [62]. Men’s Sheds provide an unstructured environment for male fellowship and valuable activities [63]. In other words, Men’s Sheds have a positive impact on the health and well-being of older men by providing a space where valuable activities take place [22]. As men tend to be less involved in community activities than women, Men’s Sheds are a place where older men are more likely to participate in social activity in communities.” |
| 3 | Page 6, line 93. The term “examine” seems to be too strong in the qualitative study. Usually, this term is used in the quantitative study. It would be better if you select an alternative verb. | This expression was revised as follows:  Page 6, line 104  Before revising: “Therefore, we asked the following research question to examine support….”  After revising: “Therefore, we asked the following research question to explore support….” |
| 4 | Pages 6-7, definitions of terms. If you talk about the definitions in this study, this information should be in the Methods section. | Following your suggestion, the “Definitions of terms” section was moved to the Methods section (page 6, line 109 – page 7, line 131). |
| 5 | Page 7, lines 112-118. You showed the levels of social activities. Did you evaluate the participants using these levels? If yes, how did they affect the extracted categories? You could show this information in the table 1. If no, I do not know why the authors introduced this information here. | This definition was needed to determine what activities should be treated as a “social activity” when conducting interviews on social activities and extracting codes from interview data. Therefore, we quoted the level of social activities defined by Levasseur, Richard, Gauvin, and Raymond (2010). To explain this intention, “Data analysis” was revised as follows:  Page 14, lines 214–218  Before revising: “The first author read the transcribed interview data carefully, removed the data using natural cuts in the manuscript as cut-off points, and coded each data. Coding did not involve simple paraphrasing—it involves elevating….”  After revising: “The first author read the transcribed interview data carefully, removed the data using natural cuts in the manuscript as cut-off points, and coded each data. The participants talked about various activities, but the narratives corresponding to level 2-6 social activities defined by Levasseur, Richard, Gauvin, and Raymond [35] were coded. The coding did not involve simple paraphrasing—it involved elevating….” |
| 6 | Page 8, participants and recruitment. Why did you choose the cooking class among various social activities? Is there any possibility that older men in that cooking class are very different from ordinary older men? What do you think about their representativeness? You could, at least, mention this point as a limitation of this study. | As you suggested, the older men in this study may have different characteristics than typical older men. It has been reported that men generally do not participate in social activities very much, but the participants in this study were mainly people who had participated in local cooking classes. That is, at the time of the interview, they were interested in social activities in the community and had actually participated in them. We added this point within the limits of our study.  Page 39, line 669 – page 40, line 686  Before revising: “The results of this study may have transferability to other older adults whose social lives mostly revolve around their workplaces.  Older male rural farmers were reported to have used two strategies to adapt to the retirement process: “gradual transition-tapering off” and “maintaining connections” [63].”  After revising: “The results of this study may have transferability to other older adults whose social lives mostly revolve around their workplaces.  Older men are less likely than women to participate in community social activities [17,18], and older men are reported to be less likely to participate in community social programs [19]. In contrast, the participants in this study were recruited from community cooking classes. Therefore, the results of this study should be understood as the experience of older men who are interested in and have participated in community social activities. However, two participants who were negative cases and were recruited outside of community cooking classes tended to have less social activity than the other 13 participants. One of the two said that he had put social activities on the back burner because of “maintaining stable family relations,” while the other said that he had to give up his participation in social activities because of functional impairments. Because this study aimed to explore the meaning and value of social activities, the focus was on the experience of older men who were engaging in social activities. Further research should explore the experience of older men who do not actively engage in social activities.  Older male rural farmers were reported to have used two strategies to adapt to the retirement process: “gradual transition-tapering off” and “maintaining connections” [71].” |
| 7 | Pages 8-9, lines 138-144. You added two community-dwelling older adults who needed assistance. Were they treated as negative cases in this study? If yes, I think that you do not need to include them in table 1. Or, they were treated as the participants under the different criteria? If you intended to analyze older adults with various conditions, it would be better to change the methodology of recruitment. | As you mentioned, the recruitment method was not adequate for analyzing older adults with various conditions. The focus of this study was on older men who participated in social activities, and the two persons who needed assistance joined this study as negative cases, which we believe enhanced the study trigger and trustworthiness. Therefore, following your suggestion, in Table 1, we deleted the information of the two persons requiring assistance. Please check Table 1 for the changes.  In addition, “Participants and Recruitment” was revised as follows:  Page 9, line 165– page 10, line 175  Before revising: “As discussed above, participation in social activities is beneficial in preventing functional impairment. In contrast, analysis suggests that functional impairments may alter the experience of participating in social activities. Therefore, for theoretical sampling, we included older men from the same community who needed assistance. We explained the purpose of the study and the inclusion criteria to the organizers at the Community Comprehensive Care Centre in this ward and asked them to introduce older men in need of assistance. Two older men who needed assistance agreed to cooperate. Typical sample sizes for grounded theory research range from 10 to 60 persons [40]; the participants in this study were 15 older men. Participants’ characteristics are shown in Table 1. Note that all the participants’ names are pseudonyms.”  After revising: “As discussed above, participation in social activities is beneficial in preventing functional impairment. In contrast, analysis suggests that functional impairments may alter the experience of participating in social activities. Therefore, to improve study rigor and trustworthiness, we included older men from the same community who needed assistance as negative cases. We explained the purpose of the study and the inclusion criteria to the organizers at the Community Comprehensive Care Centre in this ward and asked them to introduce us to older men in need of assistance. Two older men who needed assistance agreed to cooperate. Typical sample sizes for grounded theory research range from 10 to 60 persons [39]; 15 older men participated in this study. The characteristics of 13 participants recruited from cooking classes are shown in Table 1. Note that all the participants’ names are pseudonyms.”  Moreover, “Data analysis” was revised as follows:  Page 15, lines 230–239  Before revising: “The first author first categorized the interview data for five participants and modified it until consent was obtained from the three other collaborators. Next, the data for 10 participants were categorized based on the concepts drawn from the data of the five participants and revised them again until consent was obtained from the three other collaborators. From then on, each data for one participant were added until all 15 participants were completed. Variations were discovered starting from the twelfth participant’s data; but nothing new was added at the level of the conceptualized category. This led the researchers to conclude that theoretical saturation had been reached.”  After revising: “The first author initially categorized the interview data for five participants and modified it until consent was obtained from the three other collaborators. Next, the data for 10 participants were categorized based on the concepts drawn from the data of the five participants and revised them again until consent was obtained from the three other collaborators. Two of the 10 patients needed assistance were negative cases and were compared with the narratives of the other participants. From then on, each data for one participant were added until all 15 participants were completed. Variations were discovered starting from the twelfth participant’s data; but nothing new was added at the level of the conceptualized category. This led the researchers to conclude that theoretical saturation had been reached.”  Furthermore, “Study rigor and trustworthiness” was revised as follows:  Page 15, line 244 – page 16, line 251  Before revising: “Community-dwelling older men, including the research participants, attended a social gathering for preventive care where the findings of this study were shared. A member check was also conducted. One interesting comment was that, according to participants, the results were accurate in describing their vague feelings.”  After revising: “Community-dwelling older men, including the research participants, attended a social gathering for preventive care where the findings of this study were shared. A member check was also conducted in a community gathering where participants took part in. The researchers explained the results of the analysis, including the participants’ key narratives, and received feedback from the participants. One interesting comment was that, according to participants, the results were accurate in describing their vague feelings. In addition, negative cases [36] were added to the analysis and compared with other participants.”  In result section, the following sentences were revised:  Page 16, lines 256–257  Before revising: “Fifteen older men, including two people in need of support, participated.”  After revising: “Fifteen older men, including two people in need of support as negative cases, participated.”  Page 19, lines 295–297  Before revising: “Tanaka was repeatedly hospitalized and had to turn over his paint shop to his son. “I feel like I’m being told to resign, that I’m being given an *indou* by my disease,” said Tanaka, still unable to forget his work.”  After revising: “Tanaka, a negative case, was repeatedly hospitalized and had to turn over his paint shop to his son. “I feel like I’m being told to resign, that I’m being given an *indou* by my disease,” said Tanaka, still unable to forget his work.”  Page 25, line 413 – page 26, line 415  Before revising: “Suzuki, who needed to take care of his wife who had lost most of her sight, said he let go of low-priority social activities because, “*I have already done them.*””  After revising: “Suzuki, who was a negative case and needed to take care of his wife who had lost most of her sight, said he let go of low-priority social activities because, “*I have already done them.*””  We believe that further research is needed on the experience of older men who do not actively participate in social activities, such as these two men. This was added to the “Limitations and further research” section We would like you to check our response to your suggestion No. 6 regarding this correction. |
| 8 | Pages 9-11, table 1. You have the information regarding years after retirement and family composition. Did they affect the participants’ social participation? If yes, how? | A previous study indicated that retirement is a process rather than an event (Atchley, 1976, Genoe et. al., 2018). Therefore, we asked participants how many years they had been retired. Although the passage of time may be a factor in promoting adaptation to the retirement process, the results of our analysis showed that the number of years after retirement was not a factor that characterized the value and meaning of social activities. However, we believe that retirement age is one factor in understanding the characteristics of this group of participants.  In contrast, family composition was closely related to the content of the category “maintaining stable family relations.” Family members living together influenced participants to engage or not engage in social activities, while the impact of family members living far away on the participants’ social activities was very limited. In this regard, we revised the results as follows:  Page 25, line 401–405  Before revising: “In “maintaining stable family relationships,” some participants talked about using social activities to maintain a moderate distance between themselves and family members, and forging good family relationships. Sasaki, who used to teach in a university….”  After revising: “In “maintaining stable family relationships,” some participants talked about using social activities to maintain a moderate distance between themselves and family members and forging good family relationships. Family members living in remote areas rarely affected the social activities of participants. In contrast, cohabiting family members often influenced participants’ engagement in social activities. Sasaki, who used to teach in a university ….” |
| 9 | Page 15, line 213. How was a member check conducted? | Member check was performed at a community gathering that the participants attended. The researchers explained the results of the analysis, including the participants’ key narratives, and received feedback from the participants.  We added the explanation of the Member check as follows:  Page 16, lines 246–251  Before revising: “A member check was also conducted. One interesting comment was that, according to participants, the results were accurate in describing their vague feelings.”  After revising: “A member check was also conducted in a community gathering that participants took part in. The researchers explained the results of the analysis, including the participants’ key narratives, and received feedback from the participants. One interesting comment was that, according to participants, the results were accurate in describing their vague feelings.” |
| 10 | Page 16, line 245. What do you mean by “using” in the category “using and obtaining health through social activity”? Can using health and obtaining health be in the same category? The name of this category seemed to be a different nature compared with other categories. | This category was one of the most difficult to translate from Japanese to English. We could not find the right English expression.  Five values of social activity for participants were identified in this study. Basically, participants engaged in social activities because these activities had the values expressed in the five categories.  However, the category “using and obtaining health through social activity” included two concepts related to “health.” In this category, participants simultaneously told us that they were able to engage in social activities because they were healthy, and that they were able to maintain and improve their health because they engaged in social activities. In other words, only this category included value as a resource for engaging in social activities as well as value as a possible reward for engaging in social activities. Therefore, as you mentioned, the nature of this category was different from other categories.  Therefore, the category name was revised as follows:  Entire manuscript  Before revising: “using and obtaining health through social activity”  After revising: “health as a resource and reward for social activities”  In addition, we added the explanation in the Results section that only this category had different characteristics as follows:  Page 19, lines 286–292  Before revising: “First, as stated in “using and obtaining health through social activity,” health is both a capital for participating in social activity and a value obtained from participating in social activity. Yoshida said….”  After revising: “First, as stated in “health as a resource and reward for social activities,” health is both capital for participating in social activity and a value obtained from participating in social activity. In the other four categories, there was a relationship where participants engaged in social activities because of the value represented by those categories. In contrast, this category included value as a resource for engaging in social activities as well as value as a possible reward for engaging in social activities. Yoshida said….” |
| 11 | Pages 24-25, storyline. The authors mentioned two components in the social-life box: temporal void and significance void. I do not know if they come from the study results. Which category belongs to which void? I understand that one of the aims of a qualitative study is developing a hypothesis. However, this social-life box story seems to be a logical leap to me. In my opinion, this story did not explain the detailed interactions or hierarchy among categories sufficiently. For example, was there any participant who were reluctant to participate in the cooking class at the beginning? For older men, a cue of social participation maybe not always active. It would be more interesting if you show the complex interactions among categories. | To answer the following two questions, we reconsidered the relationship between categories: 1) Which categories belong to two components in the social-life box: temporary void and significance void? 2) What are the detailed interactions and hierarchies among categories?  Corbin and Strauss (2015) recommend the use of paradigm as an analytical strategy for theorizing. There are three elements to the paradigm: conditions, actions-interactions, and consequences or outcomes. These elements could facilitate understanding of the structure and processes of focused phenomena.  Therefore, the relationships among the categories extracted in this study were re-analyzed using the paradigm technique, and the description of the story line was modified as follows:  Page 27, line 447 – page 29, line 473  Before revising: “As mentioned above, the core category—“filling my social-life box”—was created tying together these eight categories. After retirement, the social-life box of participants had a temporal void (quantitative aspect) and a social significance void (qualitative aspect). Through filling this void with social activities having the following five values, participants achieved “fulfilling social life”: “using and obtaining health through social activity,” “feeling I am still useful,” feeling that something is my responsibility,” “feeling of time well spent,” and “finding interest through activities.” However, “maintaining stable family relationships” and “maintaining safety and peace in the community” were sometimes given priority over “fulfilling social life.” Participants engaged in social activities for “maintaining stable family relationships” and “maintaining safety and peace in the community,” but to prioritize them, they sometimes closed their social-life box, and abandoned social activities.”  After revising: “As mentioned above, the core category—“filling my social-life box”—was created using eight aforementioned categories. If the participants’ lives were likened to a “box,” two conditions were found to lead the participants’ to engage in social activities. The first condition was that the social activity had one of the following five values: “health as a resource and reward for social activities,” “feeling I am still useful,” feeling that something is my responsibility,” “feeling of time well spent,” and “finding interest through activities.” The second condition was that the participants had a temporal void in their life box or felt that it might occur in the future. As described in the categories of “maintaining stable family relations” and “maintaining safety and peace in the community,” the participants’ life boxes did not have room for social activities in situations where they had other activities to prioritize. To maintain control over their lives, participants used strategies to stop or abandon social activities. As consequences of their engagement in social activities, participants reached “fulfilling social life,” “maintaining stable family relations,” and “maintaining safety and peace in the community.” However, as mentioned above, the latter two categories also served as prerequisites for engaging in social activities. In addition, the participants realized the value of these activities more and more by engaging in valuable social activities, and they strengthened their engagement in these activities and pursued engagement in further activities.”  Fig 1 was partially revised in accordance with this revision. Please see Fig 1.  In addition, we revised the Abstract and Conclusion sections as follows:  Abstract (page 2, line 19 – page 3, line 53)  Before revising: “The grounded theory approach was used for the analysis. We identified that the participants associated the following five values with social activities: “using and obtaining health through social activity,” “feeling I am still useful,” “feeling that something is my responsibility,” “feeling of time well spent,” and “finding interest through interactions.” In addition, socially fulfilling a participant’s life and maintaining the peace of the family and neighborhood were extracted as the meanings of social activities. Finally, the core-category “filling social-life box” was derived. The participants experienced a time void (quantitative aspect) and a social significance void (qualitative aspect) after retirement. They fulfilled their social needs by filling the void with social activities having the aforementioned five values. Participants engaged in social activities as a strategy to adapt to post-retirement changes. Social activities could fill the void created in participants’ “social-life box” after retirement in terms of not only time but also meaning. When examining social activities that are comfortable for older men in community services, it may be useful to consider the aforementioned five values.”  After revising: “The grounded theory approach was used for the analysis. As a result, the core category—“filling my social-life box” — was extracted. Two conditions influenced participants’ engagement in social activities. The first condition was that the social activity had one of the following five values: “health as a resource and reward for social activities,” “feeling I am still useful,” feeling that something is my responsibility,” “feeling of time well spent,” and “finding interest through activities.” The second condition was that the participants had a temporal void in their life box or felt that it might occur in the future. The participants’ life boxes did not have room for social activities in situations where they had to prioritize other activities. Moreover, engaging in social activities had three meanings for the participants: “fulfilling social life,” “maintaining stable family relationships,” and “maintaining safety and peace in the community.” When considering community social activities in which older men can feel comfortable, it would be useful to incorporate social activities into their life boxes in advance and to add the above-mentioned five values to the activities.”  Conclusion (page 41, line 705 – page 42, line 736)  Before revising:  “**Conclusion**  This study explored the values and meanings of social activities for retired older men who live in an urban area of Japan. The core category—“filling my social-life box”—was created tying together these eight categories. After retirement, the social-life box of participants had a temporal void (quantitative aspect) and a social significance void (qualitative aspect). Through filling this void with social activities having the following five values, participants achieved “fulfilling social life”: “using and obtaining health through social activity,” “feeling I am still useful,” “feeling that something is my responsibility,” “feeling of time well spent,” and “finding interest through activities.” When considering the social activities that older urban retired men are interested in and likely to participate in, these five values can be a clue.  However, “maintaining stable family relationships” and “maintaining safety and peace in the community” were sometimes given priority over “fulfilling social life.” Participants engaged in social activities for “maintaining stable family relationships” and “maintaining safety and peace in the community,” but to prioritize them, they sometimes closed their social-life box, and abandoned social activities. Therefore, in situations where a peaceful life in a family or neighborhood is threatened, it may be useful to help create enough margins ahead of time to incorporate social activities into the life box.”  After revising:  “**Conclusion**  This study explored the values and meanings of social activities for retired older men who live in an urban area of Japan. The core category—“filling my social-life box”—was created using eight aforementioned categories. As consequences of their engagement in social activities, participants reached “fulfilling social life,” “maintaining stable family relations,” and “maintaining safety and peace in the community.” In addition, the participants realized the value of these activities more and more by engaging in valuable social activities, and they strengthened their engagement in these activities and pursued engagement in further activities.  There were two conditions for participants to engage in social activities. The first condition was that the social activity had one of the following five values: “health as a resource and reward for social activities,” “feeling I am still useful,” feeling that something is my responsibility,” “feeling of time well spent,” and “finding interest through activities.” When considering the social activities that older urban retired men are interested in and likely to participate in, these five values can be a clue.  The second condition was that the participants had a temporal void in their life box or felt that it might occur in the future. As described in the categories of “maintaining stable family relations” and “maintaining safety and peace in the community,” the participants’ life boxes did not have room for social activities in situations where they had other activities to prioritize. To maintain control over their lives, participants used strategies to stop or abandon social activities. Therefore, in situations where a peaceful life in a family or neighborhood is threatened, it may be useful to help create enough margins ahead of time to incorporate social activities into the life box. |
| 12 | 12. Fig 1. The sharpness is not enough. | Using the Preflight Analysis and Conversion Engine (PACE) digital diagnostic tool, we revised Fig 1. We would like you to check Fig 1. If you click on “Click here to access/download;Figure;Fig 1.tif” on the top right corner on the page of Fig 1 in the PDF file, you will see the Tiff file version. |

**Responses and revisions regarding the suggestions from reviewer: 2**

| **No.** | **Suggestions** | **Responses and revisions** |
| --- | --- | --- |
| 1 | The authors have indicated they have used Grounded theory as the methodology, but they also need to explain the epistemology (e.g. constructionism) and theoretical perspective (e.g. symbolic interactionism) that framed their study. This will help to justify their choice of grounded theory to address their research question, and situate their research more broadly. | The explanation of interactionism and pragmatism was added as a theoretical premise in “Design and context” of Method section.  **Design and context** (Page 8, line 132 – page 9, line 152)  Before revising: “This study utilized the grounded theory approach—a qualitative methodology aimed at building a theory rooted in data that allows researchers to be flexible and allow additional data as needed to saturate the resulting categories [39].”  After revising: “This study utilized the grounded theory approach—a qualitative methodology aimed at building a theory rooted in data that allows researchers to be flexible and allow additional data as needed to saturate the resulting categories [36]. This study, as well as the grounded theory approach of Corbin and Strauss, was based on the theoretical premises of interactionism and pragmatism which is greatly influenced by John Dewey and George Mead [36].  According to Blumer [37], interactionism refers to a form of interaction that occurs between people. This theory holds that humans are not just organisms that respond, but living organisms that act, and that not only interpret responses to factors that interact with them, but also create actions based on what they consider. In other words, it emphasizes that actions are not viewed as being evoked inside the actor by outside, but as being created by the actor. In addition, according to Dewey [38], knowledge comes from acting or interacting with self-reflective beings. Meaning is captured, generated, and exploited through experience and never brought in from outside sources beyond reality [38]. Furthermore, there is no binomial conflict for pragmatists, and it is impossible to remove emotions from actions, and actions have an emotional aspect [38].  This study aimed to understand the value and meaning of older men in social activities, how they engage in social activities, and the phenomenon from the viewpoint of those who act. Interactionism and pragmatism were used as theoretical premises to provide a perspective for understanding, interpreting, and redefining the empirical world of the actors in analyzing their narratives from their point of view.” |
| 2 | Please clarify if the data analysis conducted manually or via a program such as nVivo. | All data analysis was done manually. “Data analysis” was revised as follows:  Page 15, lines 227–229  Before revising: “In all of these analytical processes, researchers repeatedly used questioning, making comparison, and memo writing as analytical strategies [39].”  After revising: “In all of these analytical processes, researchers repeatedly used questioning, making comparisons, and memo writing as analytical strategies [36]. All data analysis was conducted manually.” |
| 3 | It would also be helpful to provide a table that documents how one or more of the categories were derived. This would help to more clearly distinguish some of the categories. In particular, the ‘values’ category of ‘feeling they are still useful’ has a strong overlap with the ‘meanings’ category of ‘fulfilling social life’ – particularly in relation to ‘social significance’. When looking at these different categories, the distinction between values and meanings seems somewhat artificial, and tends to obscure the real relevance of the data. | As you suggested, we added a table listing a core category, categories, and subcategories to show how the categories were derived. Please refer to Table 3 in the main manuscript.  Page 17, line 270 – page 18, line 276  Before revising: “Furthermore, the core category “filling my social-life box” was created, which tied together these eight categories. The relationship between these categories was represented by a conceptual diagram illustrating the structure of quantitative/qualitative fulfilment in retirement life using social activity among retired older men living in an urban area of Japan (Fig 1).”  After revising: “Furthermore, the core category “filling my social-life box” was created, which tied together these eight categories. Table 3 shows the list of the core category, categories, and subcategories.  [Insert Table 3 here]  In addition, the relationship between these categories was represented by a conceptual diagram illustrating the structure of quantitative/qualitative fulfilment in retirement life using social activity among retired older men living in an urban area of Japan (Fig 1).” |
| 4 | 4. The identification of the core category of ‘filling a social-life box’ provides an interesting and novel way of interpreting the data, but I’m not convinced that it adequately reflects the value and meaning of social activities for the participants, nor that it accurately conveys how social activities are implicated in older men’s health and well being. | Reviewer 1 also raised the following questions about the descriptions of the core category and storyline in the suggestion No. 11: 1) Which categories belong to two components in the social-life box: temporary void and significance void? and 2) What are the detailed interactions and hierarchies among categories?  We reconsidered the relationships among the eight categories identified as the values and meanings of social activities for the participants.  Corbin and Strauss (2015) recommend the use of paradigm as an analytical strategy for theorizing. There are three elements to the paradigm: conditions, actions-interactions, and consequences or outcomes. These elements could facilitate understanding of the structure and processes of focused phenomena.  Therefore, the relationships among the categories extracted in this study were re-analyzed using the paradigm technique, and the description of the story line was modified as follows:  Page 27, line 447 – page 29, line 473  Before revising: “As mentioned above, the core category—“filling my social-life box”—was created tying together these eight categories. After retirement, the social-life box of participants had a temporal void (quantitative aspect) and a social significance void (qualitative aspect). Through filling this void with social activities having the following five values, participants achieved “fulfilling social life”: “using and obtaining health through social activity,” “feeling I am still useful,” feeling that something is my responsibility,” “feeling of time well spent,” and “finding interest through activities.” However, “maintaining stable family relationships” and “maintaining safety and peace in the community” were sometimes given priority over “fulfilling social life.” Participants engaged in social activities for “maintaining stable family relationships” and “maintaining safety and peace in the community,” but to prioritize them, they sometimes closed their social-life box, and abandoned social activities.”  After revising: “As mentioned above, the core category—“filling my social-life box”—was created using eight aforementioned categories. If the participants’ lives were likened to a “box,” two conditions were found to lead the participants’ to engage in social activities. The first condition was that the social activity had one of the following five values: “health as a resource and reward for social activities,” “feeling I am still useful,” feeling that something is my responsibility,” “feeling of time well spent,” and “finding interest through activities.” The second condition was that the participants had a temporal void in their life box or felt that it might occur in the future. As described in the categories of “maintaining stable family relations” and “maintaining safety and peace in the community,” the participants’ life boxes did not have room for social activities in situations where they had other activities to prioritize. To maintain control over their lives, participants used strategies to stop or abandon social activities. As consequences of their engagement in social activities, participants reached “fulfilling social life,” “maintaining stable family relations,” and “maintaining safety and peace in the community.” However, as mentioned above, the latter two categories also served as prerequisites for engaging in social activities. In addition, the participants realized the value of these activities more and more by engaging in valuable social activities, and they strengthened their engagement in these activities and pursued engagement in further activities.  Fig 1 was partially revised in accordance with this revision. We would also like you to check Fig 1.  In addition, we revised the Abstract and Conclusion sections as follows:  Abstract (page 2, line 19 – page 3, line 53)  Before revising: “The grounded theory approach was used for the analysis. We identified that the participants associated the following five values with social activities: “using and obtaining health through social activity,” “feeling I am still useful,” “feeling that something is my responsibility,” “feeling of time well spent,” and “finding interest through interactions.” In addition, socially fulfilling a participant’s life and maintaining the peace of the family and neighborhood were extracted as the meanings of social activities. Finally, the core-category “filling social-life box” was derived. The participants experienced a time void (quantitative aspect) and a social significance void (qualitative aspect) after retirement. They fulfilled their social needs by filling the void with social activities having the aforementioned five values. Participants engaged in social activities as a strategy to adapt to post-retirement changes. Social activities could fill the void created in participants’ “social-life box” after retirement in terms of not only time but also meaning. When examining social activities that are comfortable for older men in community services, it may be useful to consider the aforementioned five values.”  After revising: “The grounded theory approach was used for the analysis. As a result, the core category—“filling my social-life box” — was extracted. Two conditions influenced participants’ engagement in social activities. The first condition was that the social activity had one of the following five values: “health as a resource and reward for social activities,” “feeling I am still useful,” feeling that something is my responsibility,” “feeling of time well spent,” and “finding interest through activities.” The second condition was that the participants had a temporal void in their life box or felt that it might occur in the future. The participants’ life boxes did not have room for social activities in situations where they had to prioritize other activities. Moreover, engaging in social activities had three meanings for the participants: “fulfilling social life,” “maintaining stable family relationships,” and “maintaining safety and peace in the community.” When considering community social activities in which older men can feel comfortable, it would be useful to incorporate social activities into their life boxes in advance and to add the above-mentioned five values to the activities.  Conclusion (page 41, line 705 – page 42, line 736)  Before revising:  “**Conclusion**  This study explored the values and meanings of social activities for retired older men who live in an urban area of Japan. The core category—“filling my social-life box”—was created tying together these eight categories. After retirement, the social-life box of participants had a temporal void (quantitative aspect) and a social significance void (qualitative aspect). Through filling this void with social activities having the following five values, participants achieved “fulfilling social life”: “using and obtaining health through social activity,” “feeling I am still useful,” “feeling that something is my responsibility,” “feeling of time well spent,” and “finding interest through activities.” When considering the social activities that older urban retired men are interested in and likely to participate in, these five values can be a clue.  However, “maintaining stable family relationships” and “maintaining safety and peace in the community” were sometimes given priority over “fulfilling social life.” Participants engaged in social activities for “maintaining stable family relationships” and “maintaining safety and peace in the community,” but to prioritize them, they sometimes closed their social-life box, and abandoned social activities. Therefore, in situations where a peaceful life in a family or neighborhood is threatened, it may be useful to help create enough margins ahead of time to incorporate social activities into the life box.”  After revising:  “**Conclusion**  “This study explored the values and meanings of social activities for retired older men who live in an urban area of Japan. The core category—“filling my social-life box”—was created using eight aforementioned categories. As consequences of their engagement in social activities, participants reached “fulfilling social life,” “maintaining stable family relations,” and “maintaining safety and peace in the community.” In addition, the participants realized the value of these activities more and more by engaging in valuable social activities, and they strengthened their engagement in these activities and pursued engagement in further activities.  There were two conditions for participants to engage in social activities. The first condition was that the social activity had one of the following five values: “health as a resource and reward for social activities,” “feeling I am still useful,” feeling that something is my responsibility,” “feeling of time well spent,” and “finding interest through activities.” When considering the social activities that older urban retired men are interested in and likely to participate in, these five values can be a clue.  The second condition was that the participants had a temporal void in their life box or felt that it might occur in the future. As described in the categories of “maintaining stable family relations” and “maintaining safety and peace in the community,” the participants’ life boxes did not have room for social activities in situations where they had other activities to prioritize. To maintain control over their lives, participants used strategies to stop or abandon social activities. Therefore, in situations where a peaceful life in a family or neighborhood is threatened, it may be useful to help create enough margins ahead of time to incorporate social activities into the life box. |
| 5 | My main concern is that in striving to derive theory through data, the authors have overlooked a significant body of literature that is very relevant to the issues raised in their study. As such, they have not adequately situated their theory of ‘filling a social-life box’ in the literature – this is a key requirement of grounded theory studies. Moreover, I get a strong sense that the analysis has ‘reinvented the wheel’ – that is, the authors have developed a theory on the values and meanings of social activities among older retired men that aligns closely with relevant literature and theory, but have not adequately acknowledged that literature, or attempted to situation their findings within that literature. In particular, the authors should consider how their findings relate to literature on social capital and social support among older men. For example, their discussion on how participants engaged in social activities to ‘maintain safety and peace in the community’ relates closely to the concept of social capital, and the notion that community relationships can be leveraged as a ‘commodity’ to draw on in times of stress and need. Indeed, Matsumoto’s comment about why human connections are important reflective on the value of social capital. Significantly, social capital works at both the individual and community level, and can act to support the health of men who are not socially connected, as well as those who do have strong social connections. Other aspects of social capital that are relevant to the findings of this study include the notion of bonding and bridging capital – this relates to Yamamoto’s comment on community connection. Therefore, this paper would be considerably strengthened by integrating a discussion on how their findings link to the literature on social capital in the Discussion section. | While we discussed the adaptation to life after retirement from the perspective of the participants in this study, we did not consider the social significance of social activities. As you suggested, the category “maintain safety and peace in the community” was not only meaningful at the individual level, but also at the community level; social capital is created in the community where the participants live.  Therefore, we reviewed previous studies on social capital and added the following discussion about the relationship with the results of this study:  Page 34, line 567 – page 35, line 594  Before revising: “This suggests that the social activities that older people want to perform depend not only on gender but also on one’s previous lifestyle.”  After revising: “This suggests that the social activities that older people want to perform depend not only on gender, but also on one’s previous lifestyle.  The category “maintain safety and peace in the community” could be redefined at the community level as a concept of social capital. To date, several classification systems for social capital have been suggested of which the concept of bonding and bridging social capital has credence [56]. According to Szreter and Woolcock [57], bonding social capital refers to aspects of “inward-looking” social networks that reinforce exclusive identities and homogeneous groups (e.g., age, ethnicity, and social class). In contrast, bridging social capital refers to “outward-looking” social networks across different social and ethnic groups that do not necessarily share similar identities. For “maintain safety and peace in the community,” participants said that they sought to foster both bonding and bridging social capital among homogeneous/heterogeneous groups of age and social class in the neighborhood. In particular, participants emphasized that they should avoid crossing certain boundaries when connecting with neighbors. This might be a strategy for the participants to build bridging social capital smoothly.  In addition, Saito et al. [58] identified the following three subscales for community-level social capital: civic participation, social cohesion, and recipient. The social activity for “maintain safety and peace in the community” carried out by the participants of this study could be significant to foster social cohesion, which is a factor strongly related to trust, community trust, and attachment [58]. Nine of the participants in this study were directors of neighborhood associations, which may have been influenced by their high interest in improving social cohesion. Noguchi et al. [59] reported that living in communities with high social cohesion was associated with a decreased incidence of functional impairment in older men. Moreover, Amemiya et al. [60] showed that in communities with high social cohesion, older men who perceived that their communities’ social cohesion was high showed greater functional ability improvement than men who perceived it to be low. The social activities for “maintain safety and peace in the community” could have both self- and community-interest for the participants.”  In accordance with the revisions of No. 4 and No. 5, the subheadings of the Discussion section were revised as follows:  Page 29, lines 482–484  Before revising: “Filling the temporal and significance void in the social-life box”  After revising: “Characteristics of the meanings in social activities for older urban men” |
| 6 | Likewise, the categories identified in this study such as ‘feeling that something is my responsibility’ and ‘feeling they are still useful’ have direct relevance to the literature on meaning and purpose among older people. Again, this paper would be considerably strengthened by integrating a discussion on how their findings link to existing literature in this area. | Following your suggestion, we reviewed literature on measuring and purpose among older people. In relation to the results of this study, the following discussion was added:  Page 31, line 511– page 32, line 529  Before revising: “Social activity may have been a useful tool for men in addressing the quantitative void that appeared after retirement.  Maintaining a peaceful life within the family and in the neighborhood was the most important and basic aspect of participants’ post-retirement lives….”  After revising: “Social activity may have been a useful tool for men in addressing the quantitative void that appeared after retirement.  In addition, “filling social-life box” may have provided participants with a real sense of meeting in life (MiL). Bartrés-Faz et. al. [50] describes MiL based on the following three components: purpose in life, sense of coherence, and engagement with life. Among them, engagement with life refers to the individual’s evaluation of how important, worthwhile, and inherently valuable their life feels and the sense of having a life worth living. Bartrés-Faz et. al. [50] further notes that this component covers the relevance of perceived significance and fulfillment of one’s life and the importance of engaging in valued and meaningful activities. Participants in this study achieved “filling social-life box” through engaging in social activities valued from five perspectives. This could be similar to achieving engagement with life through engaging in valued and measurable activities. Furthermore, of the five values of social activities, “feeling I am still useful” and “feeling that something is my responsibility” may be related to the realization of purpose in life, which is the component of the MiL. Purpose in life refers to having a strong sense of meaning and future-oriented goals in life [50]. The participants’ sense of purpose in life might be strengthened by engaging in social activities where they could feel as if they are still useful and that something is their responsibility.”  Maintaining a peaceful life within the family and in the neighborhood was the most important and basic aspect of participants’ post-retirement lives….” |
| 7 | There are some problems with English grammar – please carefully proof read the paper. | The entire manuscript was revised to solve grammatical problems and improve readability through detailed English proofreading. These revisions are also colored in red in the main document. |
