# Supplementary material for: The values and meanings of social activities for older urban men after retirement
Source: PLoS One. 2020 Nov 25;15(11):e0242859. doi: 10.1371/journal.pone.0242859 (PMC7688116; doi:10.1371/journal.pone.0242859)
Supplement: Attachment — Submitted filename: Response to Reviewers.docx [file pone.0242859.s002.docx]

Dear Reviewers,

Thank you very much for providing us with your insights regarding our manuscript. We have revised our paper accordingly, as shown point-by-point below. The additions are marked in red and deleted text is tracked via the “Track Changes” function in Microsoft Word. The page and line numbers in this document are based on the main manuscript with the revision history and revisions shown in-line.

We would appreciate it if you reviewed our manuscript again. Thank you very much.

**Responses and revisions regarding the suggestions from reviewer: 1**

| **No.** | **Suggestions** | **Responses and revisions** |
| --- | --- | --- |
| 1 | Thank you for revising the manuscript based on my comments. To enhance the quality of your article, I would like to suggest to you for some minor revisions as follows.  1. Page 1, the authors’ name. The last letter of the authors’ family name may be unnecessary. Onishib could be Onishi, Saekic could be Saeki, and Hiranob could be Hirano. Please ensure the accuracy of each expression very carefully. | I am sorry for this careless mistake. Thank you for carefully reviewing the manuscript.  I revised it as follows:  Page 1, line 6  Before revising:  “Risa Takashima^1^, Ryuta Onishib^2^, Kazuko Saekic^3^, Michiyo Hiranob^2*^”  After revising:  “Risa Takashima^1^, Ryuta Onishi~~b~~^2^, Kazuko Saeki~~c~~^3^, Michiyo Hirano~~b~~^2*^” |
| 2 | 2. Page 2, line 25. The verb “examine” sounds too strong in the qualitative study. You could change this to another verb if you agree. | Following your advice, we revised “examined” to “explored” as shown below.  Page 2, line 24–27  Before revising: “We examined the values and meanings of social activities for retired older men living in an urban area of Japan to examine support using social activities that are more suitable for them.”  After revising: “We explored the values and meanings of social activities for retired older men living in an urban area of Japan to understand support using social activities that are more suitable for them.”  In addition, “examined” used in the Methods were also revised to “explored”.  Page 13, lines 213–215  Before revising: “Following this, the relationships between top-level categories were examined using a diagram or a visual device that depicts the relationships between analytical concepts [36].”  After revising: “Following this, the relationships between top-level categories were explored using a diagram or a visual device that depicts the relationships between analytical concepts [36].” |
| 3 | 3. This time, you emphasized “filling my social-life box” as the core category. However, this change brought me a feeling of strangeness. “Filling my social-life box” seems to be a hypothetical concept rather than a category extracted from the data. I understand the importance of this concept in your study line. However, your main focus should be to explore the values and meanings of social activities as you mentioned in the introduction. I recommend you to treat “filling my social-life box” just as an idea of your interpretation. Otherwise, readers would have an impression of a logical leap. | Following your advice, we have focused on the values and significance of social activities and have treated “filling my social-life box” as a hypothetical concept to help to understand the 8 categories. Therefore, the content of the Storyline was moved to the Discussion along with Fig 1.  However, if you think that the content related to the hypothetical concept, “filling my social-life box,” and Fig 1 should be deleted after you check the revised manuscript, please let us know as we are prepared to do so.  Data analysis (page 14, lines 211–219)  Before revising: “Based on properties and dimensions, coded concepts were compared, and related concepts were categorized into higher-level concepts. Three levels of categorization were conducted after coding. Following this, the relationships between top-level categories were examined using a diagram or a visual device that depicts the relationships between analytical concepts [36]. Finally, the core category [36] was explored and integrated to link each category together, refining the final theoretical construct. A storyline was created as a technique to aid the integration [36]. In all of these analytical processes, researchers repeatedly used questioning, making comparisons, and memo writing as analytical strategies [36].”  After revising: “Based on properties and dimensions, coded concepts were compared, and related concepts were categorized into higher-level concepts. Three levels of categorization were conducted after coding. Following this, the relationships between top-level categories were explored using a diagram or a visual device that depicts the relationships between analytical concepts [36]. ~~Finally, the core category [36] was explored and integrated to link each category together, refining the final theoretical construct. A storyline was created as a technique to aid the integration [36].~~ In all of these analytical processes, researchers repeatedly used questioning, making comparisons, and memo writing as analytical strategies [36].”  Findings (page 16, lines 257–260)  Before revising: “…and “maintaining safety and peace in the community.” Furthermore, the core category “filling my social-life box” was created, which tied together these eight categories. Table 3 shows the list of the core category, categories, and subcategories.”  After revising: “…and “maintaining safety and peace in the community.” ~~Furthermore, the core category “filling my social-life box” was created, which tied together these eight categories.~~ Table 3 shows the list of ~~the core category,~~ features of categories, categories, and subcategories.”  With this revision, we deleted the column of the core category in Table 3. We would like you to see Table 3 (page 16, line 261).  Findings (page 17, line 263 – page 18, line 267)  Before revising: “In addition, the relationship between these categories was represented by a conceptual diagram illustrating the structure of quantitative/qualitative fulfilment in retirement life using social activity among retired older men living in an urban area of Japan (Fig 1).  Fig 1. Filling one’s social-life box using social activities in older urban men after retirement.”  After revising: Delete  Before revising (page 26, line 433 – page 27, line 450):  “**Storyline (Fig 1)**  As mentioned above, the core category—“filling my social-life box”—was created using eight aforementioned categories. If the participants’ lives were likened to a “box,” two conditions were found to lead the participants’ to engage in social activities.…However, as mentioned above, the latter two categories also served as prerequisites for engaging in social activities. In addition, the participants realized the value of these activities more and more by engaging in valuable social activities, and they strengthened their engagement in these activities and pursued engagement in further activities.”  After revising (page 28, line 469 – page 27, line 486): We moved the content of the Storyline to the Discussion and modified some expressions as follows:  “The concept of “filling my social-life box” might relate the eight aforementioned categories to each other. If the participants’ lives were likened to a “box,” it seems that two conditions were found to lead the participants’ to engage in social activities…. However, as mentioned above, the latter two categories also seemed to serve as prerequisites for engaging in social activities. In addition, the participants were increasingly likely to realize the value of these activities by engaging in valuable social activities; consequently, they might strengthen their engagement in these activities and pursue engagement in further activities.”  Discussion (page 27, line 453 – page 28, line 468)  Before revising: “To understand the values and meanings of social activities for retired older men who live in an urban area of Japan, it is necessary to consider the impact of retirement on their lives. Retirement creates a significant void in the lives of participants in the dimensions of time and social significance. In addition, the center of participants’ lives will shift from the workplace to the community. Engagement in social activities appears to play an important role in helping older male participants adjust to life in the community.”  After revising: “This study explored the values and meanings of social activities for retired older men who live in an urban area of Japan. Five and three categories were derived as the values and meanings that participants place on the social activities that they engage in, respectively. To understand their values and meanings of social activities for these older men, it is necessary to consider the impact of retirement on their lives. Retirement creates a significant void in the lives of participants in the dimensions of time and social significance. In addition, the center of participants’ lives will shift from the workplace to the community. Engagement in social activities appears to play an important role in helping older male participants adjust to life in the community. Moreover, this process of the adjustment may be interpreted as a hypothetical concept—“filling my social-life box”—that might link the 8 categories.  To help understand the concept of “filling my social-life box,” a conceptual diagram illustrating the structure of quantitative/qualitative fulfilment in retirement life using social activity among retired older men was created (Fig 1).  **Fig 1. Filling one’s social-life box using social activities in older urban men after retirement.**”  In conjunction with these revisions, following your advice, we have emphasized the values and meanings of social activities and removed the statement regarding “filling my social-life box” in the Abstract and the Conclusion.  Abstract (Page 2, line 29 – page 3, line 54)  Before revising:  “The grounded theory approach was used for the analysis. As a result, the core category—“filling my social-life box” — was extracted. Two conditions influenced participants’ engagement in social activities. The first condition was that the social activity had one of the following five values: “health as a resource and reward for social activities,” “feeling I am still useful,” feeling that something is my responsibility,” “feeling of time well spent,” and “finding interest through activities.” The second condition was that the participants had a temporal void in their life box or felt that it might occur in the future. The participants’ life boxes did not have room for social activities in situations where they had to prioritize other activities. Moreover, engaging in social activities had three meanings for the participants: “fulfilling social life,” “maintaining stable family relationships,” and “maintaining safety and peace in the community.” When considering community social activities in which older men can feel comfortable, it would be useful to incorporate social activities into their life boxes in advance and to add the above-mentioned five values to the activities.”  After revising:  “The grounded theory approach was used for the analysis. As a result, the following five categories were derived as the values that participants place on the social activities that they engage in: “health as a resource and reward for social activities,” “feeling I am still useful,” “feeling that something is my responsibility,” “feeling of time well spent,” and “finding interest through interactions.” In addition, the following three categories were extracted as meanings of social activities: “fulfilling social life,” “maintaining stable family relationships,” and “maintaining safety and peace in the community.” When considering the social activities that older urban retired men are interested in and likely to participate in, these five values can be considered indicators. In contrast, to maintain stable family relationships and safety and peace in the community, participants sometimes used strategies to stop or abandon social activities. Therefore, in situations where a peaceful life within a family or neighborhood is threatened, it may be useful to help set aside sufficient time and allow for psychological leeway in advance to incorporate social activities into their lives.”  Conclusion (Page 41, line 718 – page 43, line 745)  Before revising:  “This study explored the values and meanings of social activities for retired older men who live in an urban area of Japan. The core category—“filling my social-life box”—was created using eight aforementioned categories. As consequences of their engagement in social activities, participants reached “fulfilling social life,” “maintaining stable family relations,” and “maintaining safety and peace in the community.” In addition, the participants realized the value of these activities more and more by engaging in valuable social activities, and they strengthened their engagement in these activities and pursued engagement in further activities.  There were two conditions for participants to engage in social activities. The first condition was that the social activity had one of the following five values: “health as a resource and reward for social activities,” “feeling I am still useful,” feeling that something is my responsibility,” “feeling of time well spent,” and “finding interest through activities.” When considering the social activities that older urban retired men are interested in and likely to participate in, these five values can be a clue.  The second condition was that the participants had a temporal void in their life box or felt that it might occur in the future. As described in the categories of “maintaining stable family relations” and “maintaining safety and peace in the community,” the participants’ life boxes did not have room for social activities in situations where they had other activities to prioritize. To maintain control over their lives, participants used strategies to stop or abandon social activities. Therefore, in situations where a peaceful life in a family or neighborhood is threatened, it may be useful to help create enough margins ahead of time to incorporate social activities into the life box.”  After revising:  “This study explored the values and meanings of social activities for retired older men who live in an urban area of Japan. The following five categories were derived as the values that participants place on the social activities that they engage in: “health as a resource and reward for social activities,” “feeling I am still useful,” “feeling that something is my responsibility,” “feeling of time well spent,” and “finding interest through interactions.” When considering the social activities that older urban retired men are interested in and likely to participate in, these five values can be taken as indicators. In addition, the following three categories were extracted as meanings of social activities: “fulfilling social life,” “maintaining stable family relationships,” and “maintaining safety and peace in the community.” As described in the categories of “maintaining stable family relations” and “maintaining safety and peace in the community,” the participants’ lives did not have leeway for engaging in social activities in situations where they had other activities to prioritize. To maintain control over their lives, participants used strategies to stop or abandon social activities. Therefore, in situations where a peaceful life in a family or neighborhood is threatened, it may be useful to help set aside sufficient time and allow for psychological leeway to incorporate social activities into their lives.” |

In addition, the entire manuscript was revised to resolve grammatical issues and improve readability through detailed English proofreading. These revisions are shown using Track Changes.

**Responses and revisions regarding the suggestions from reviewer: 2**

| **No.** | **Suggestions** | **Responses and revisions** |
| --- | --- | --- |
| 1 | Thank you to the authors for their comprehensive response to the reviewer comments. I am satisfied with their response to my comments. There are just a few outstanding issues that need attending to:  1. Methods, Design and context section a. I don’t understand what the authors mean by the phrase – “Furthermore, there is no binomial (??) conflict for pragmatists, and it is impossible to remove emotions from actions, and actions have an emotional response” (p. 8 line 125-127) – could the authors please simplify this statement to clarify their meaning? | The word “binomial” was mistranslated, and “dualism” was the correct expression.  Corbin and Strauss (2015, p. 23), quoting Dewey (1929), describe one of the assumptions of their method as follows:  “Assumption 10. Actions has emotional aspects. To conceive of emotion as distinguishable from action is to reify those aspects of action. For us, there is no dualism. One can’t separate emotion from action; they flow together one leading into the other (Dewey, 1929).”  Based on this explanation, we revised the phrasing as follows:  Page 8, lines 137–139  Before revising: “Furthermore, there is no binomial conflict for pragmatists, and it is impossible to remove emotions from actions, and actions have an emotional aspect [38].”  After revising: “Furthermore, there is no dualism for pragmatists, and it is impossible to separate emotions from actions; they flow together with one leading into the other [38].” |
| 2 | b. Likewise, the first sentence in the next paragraph also needs rephrasing to enhance meaning – “This study aimed to understand the value and meaning of older men in social activities”. Do the authors mean “This study aimed to understand the value and meaning that older men ascribe to their social activities”? (p. 8, line 128) | Thank you for providing this concrete idea for paraphrasing. We have revised this sentence as you advised.  Page 8, lines 140–141  Before revising: “This study aimed to understand the value and meaning of older men in social activities”  After revising: “This study aimed to understand the value and meaning that older men ascribe to their social activities” |
| 3 | 2. Discussion, Characteristics of the meanings… section a. p. 30, lines 487- 488 - the sentence reads “…may be related to the realization of purpose in life, which is the component of the MiL”. I suggest this be changed to - “…may be related to the realization of purpose in life, which is a component of the MiL”. | Following your advice, we revised it as follows:  Page 31, lines 528–529  Before revising: “…may be related to the realization of purpose in life, which is the component of the MiL”  After revising: “…may be related to the realization of purpose in life, which is a component of the MiL” |
| 4 | b. P. 33, lines 541-543 – I don’t think the participants said that they sought to foster both bonding and bridging social capital! This needs to be rephrased to indicate that through their various social activities, participants appeared to be trying to build forms of bonding and bridging social capital. | As you mentioned, this expression was not appropriate. Following your advice, we revised the wording as follows:  Page 34, lines 582–586  Before revising: “For ‘maintain safety and peace in the community,’ participants said that they sought to foster both bonding and bridging social capital among homogeneous/heterogeneous groups of age and social class in the neighborhood.”  After revising: “For “maintain safety and peace in the community,” through their various social activities, participants appeared to be trying to build forms of bonding and bridge social capital.” |
| 5 | c. P. 33, line 548 – should one of Saito et al., subscales be ‘recipient’? This doesn’t seem to align with the other subscales that are listed here (civic participation and social coesion). | I am sorry for this careless mistake. As you mentioned, “recipient” was used instead of “reciprocity.” I revised it as follows:  Page 35, lines 589–590  Before revising: “In addition, Saito et al. [58] identified the following three subscales for community-level social capital: civic participation, social cohesion, and recipient.”  After revising: “In addition, Saito et al. [58] identified the following three subscales for community-level social capital: civic participation, social cohesion, and reciprocity.” |
| 6 | d. P. 33, lines 554-557 – sentence beginning “Moreover, Amemiya … showed greater functional ability improvement than men who perceived it to be low” – to help the reader understand this, it would be useful to provide an example of functional improvement. | Based on your comment, we added an explanation regarding functional ability as follows:  Page 35, lines 597–604  Before revising: “Amemiya et al. [60] showed that in communities with high social cohesion, older men who perceived that their communities’ social cohesion was high showed greater functional ability improvement than men who perceived it to be low.”  After revising: “Amemiya et al. [60] showed that in communities with high social cohesion, older men who perceived that their communities’ social cohesion was high showed greater functional ability improvement than men who perceived it to be low. In their study, functional ability was assessed through levels of disability, which were objectively assessed at the time of certification for the utilization of long-term care (LTC) services, based on nationally standardized criteria in Japan. There are seven levels of disability: Requiring Support-1 and -2 and Requiring LTC-l (partial support needed for basic activities of daily living) to LTC-5 (complete support needed for all activities of daily living).” |
| 7 | e. Pp 33-34 – last line on p. 33 and first line on p. 34 – sentence beginning “The social activities for…” – I’m not sure what the authors mean by “could have both self- and community-interest for the participants”. Could the authors please clarify what they mean by self and community interest, in the context of the broader discussion in this para on social capital? | This sentence was not well explained. The theme of “maintain safety and peace in the community” basically indicated that the participants were oriented toward the community in which they live and engaged in social activities for the benefit of the community. In this sense, we believe that the social activities represented by this theme have benefited the community (community-interest).  In contrast, the studies by Noguchi et al. and Amemiya et al. cited in this paragraph (page 35, lines 595–604) showed that communities with high social cohesion had a positive impact on the functional abilities of older men living in the community. In this sense, we thought that the social activities for “maintain safety and peace in the community” would also benefit the participants themselves (self-interest).  In order to convey this intention more clearly, the following revision was made:  Page 35, line 604 – page 36, line 607  Before revising: “The social activities for “maintain safety and peace in the community” could have both self- and community-interest for the participants.”  After revising: “The social activities for “maintain safety and peace in the community” may not only benefit the community by increasing the social cohesion but may also benefit the functional health of older men living in the community, including the participants themselves.” |

In addition, the entire manuscript was revised to resolve grammatical issues and improve readability through detailed English proofreading. These revisions are shown using Track Changes.
